# Developing and evaluating the implementation of a complex intervention: using mixed methods to inform the design of a randomised controlled trial of an oral healthcare intervention after stroke

**DOI:** 10.1186/1745-6215-12-168

**Published:** 2011-07-05

**Authors:** Marian C Brady, David J Stott, John Norrie, Campbell Chalmers, Bridget St George, Petrina M Sweeney, Peter Langhorne

**Affiliations:** 1 Nursing, Midwifery and Allied Health Professions Research Unit, Glasgow Caledonian University, Glasgow, UK; 2 Academic Section of Geriatric Medicine, Glasgow Royal Infirmary, UK; 3 Robertson Centre of Biostatistics, University of Glasgow, UK; 4 NHS Lanarkshire, Scotland, UK; 5 Dental School, University of Glasgow, UK

## Abstract

**Background:**

Many interventions delivered within the stroke rehabilitation setting could be considered complex, though some are more complex than others. The degree of complexity might be based on the number of and interactions between levels, components and actions targeted within the intervention. The number of (and variation within) participant groups and the contexts in which it is delivered might also reflect the extent of complexity. Similarly, designing the evaluation of a complex intervention can be challenging. Considerations include the necessity for intervention standardisation, the multiplicity of outcome measures employed to capture the impact of a multifaceted intervention and the delivery of the intervention across different clinical settings operating within varying healthcare contexts. Our aim was to develop and evaluate the implementation of a complex, multidimensional oral health care (OHC) intervention for people in stroke rehabilitation settings which would inform the development of a randomised controlled trial.

**Methods:**

After reviewing the evidence for the provision of OHC following stroke, multi-disciplinary experts informed the development of our intervention. Using both quantitative and qualitative methods we evaluated the implementation of the complex OHC intervention across patients, staff and service levels of care. We also adopted a pragmatic approach to patient recruitment, the completion of assessment tools and delivery of OHC, alongside an attention to the context in which it was delivered.

**Results:**

We demonstrated the feasibility of implementing a complex OHC intervention across three levels of care. The complementary nature of the mixed methods approach to data gathering provided a complete picture of the implementation of the intervention and a detailed understanding of the variations within and interactions between the components of the intervention. Information on the feasibility of the outcome measures used to capture impact across a range of components was also collected, though some process orientated uncertainties including eligibility and recruitment rates remain to be further explored within a Phase II exploratory trial.

**Conclusions:**

Complex interventions can be captured and described in a manner which facilitates evaluation in the form of exploratory and subsequently definitive clinical trials. If effective, the evidence captured relating to the intervention context will facilitate translation into clinical practice.

## Background

### Oral Health Care

Caring for one's teeth and mouth, or oral health care (OHC), is a fundamental aspect of self-care. Typically we care for our own teeth in an independent, individualised manner which requires little thought, but which reflects our individual oral health needs, preferences, standards and patterns of behaviour. Some will occasionally (if at all) seek professional intervention in the form of an annual or bi-annual visit to the dentist [1]. Therefore the components of OHC may be perceived at one level as a series of relatively simple tasks or interventions. However, where there is an acquired deficit in an individual's cognitive capacity or physical ability, as might occur following a stroke, that individual may become partially or totally reliant upon others to ensure their oral health, temporarily or on a continuing basis. Thus the potential complexity of providing or supporting OHC across a patient population with a range of abilities, with varying (or even changing) OHC needs whilst also ensuring they maintain (or attain) good oral health becomes more apparent.

### Oral Health Care after Stroke

Many individuals experience stroke related physical, cognitive, visual or sensory deficits which may make independent OHC difficult. Swallowing, chewing and oral clearance difficulties as a result of the stroke may leave food or fluid residue within the mouth for prolonged periods of time, contributing to dental decay [2]. In addition, for people with stroke related swallowing difficulties, medication may be provided in a syrup based consistency which may also inadvertently contribute to dental problems. Known side effects of medication or treatment prescribed following stroke may also impact upon oral health (for example, dry mouth, oral ulcers and stomatitis) [3]. Some individuals experiencing such stroke related challenges to maintaining oral health may also experience these difficulties in the presence of pre-existing oral health problems [1,4] further compounding oral health risks. Many patients within the stroke care setting may be partially or totally reliant upon nursing assistance to ensure their oral health [5].

### Evidence base

Before any new OHC intervention can be developed and evaluated there needs to be a strong theoretical underpinning for that intervention [6]. Undertaking a considerable programme of pre-clinical work [6] we conducted a Cochrane systematic review and found a very limited evidence base [7]. Stroke specific data from a small randomised controlled trial (RCT) of an OHC educational intervention for staff based in a nursing home setting reported positive benefits to residents' denture cleanliness (but not dental cleanliness) as a result of a staff training intervention [8,9]. Some patient subgroups were excluded - for example those with significant cognitive impairment and those who were edentulous (who had neither natural teeth nor dentures). Other more recent RCTs have examined periodontal therapy [10] or an oral decontamination gel [11], neither of which could be considered 'routine' oral health care. Two trials that did aim to evaluate complex OHC interventions investigated a ventilated post stroke population [12] and a nursing home population [13]. Both trials were problematic methodologically. The nursing home population were a highly selected group that excluded those that were unwell, cognitively incapacitated or receiving palliative care [13] - patient subgroups at high risk of oral health problems and requiring the most intensive OHC support. In contrast, the trial with the ventilated population was terminated before completion and only reported data on the incidence of pneumonia [12].

### Complex Interventions

Many interventions delivered within the stroke rehabilitation setting could be considered complex, though some are more complex than others, occurring at different points along what could be considered a spectrum of complexity [6]. The degree of complexity might be gauged on the basis of the number of; components within the intervention (and the interactions between those components); actions required from participants; actions required from those delivering the intervention; organisational levels the intervention is targeting; and outcome measures employed [6]. Others describe complexity at a systems level [14] urging careful consideration of the clinical context in which an intervention is delivered. In practice, both the intervention and the systems within which it operates are relevant thus the number of systems could also be considered in relation to the degree of complexity. For example, an OHC intervention is delivered to a patient, by staff operating as a team within a ward, which in turn is nested within a hospital, which may be further supported by external specialist dental support services (as required). For the OHC intervention to work the intervention must function within, and be supported by, the team, ward, hospital and external services.

Researchers however have often strived to simplify research questions. Reducing variability in the participants, the intervention, the delivery and/or the context and introducing consistency across these parameters with the aim of increasing internal and construct validity i.e. an efficacy study [15]. However such a narrow approach to intervention evaluation comes at the expense of external validity. As a consequence, many evaluations of OHC interventions after stroke recruited very narrow patient populations, delivered very specific oral healthcare protocols which were implemented by specialist researchers or healthcare staff that were atypical within the normal clinical setting. As a result there are limitations on the clinical relevance of the study leading to delayed translation into clinical practice [15].

In contrast, drawing on and accommodating the known components of complexity during the development and evaluation of an OHC intervention, informs the development of a clinical effectiveness study [15]. Thus a clinically feasible, adaptable OHC intervention is delivered to a heterogeneous, clinically representative post stroke population within a typical stroke healthcare setting by a typical stroke rehabilitation team. Should it prove effective, capturing data on the interactions observed within and between components of the intervention, and the systems across which the intervention is delivered, will facilitate the translation of the research into practice [15,16].

### Context

Delivery of complex, clinically embedded interventions, within a healthcare context does not occur in isolation. As such, researchers need to consider the consequences of potential interactions between the intervention and the 'system' (context in which it is delivered) in designing and evaluating the intervention [14]. Thus capturing information on contextual factors is essential in clinical research [16]. Evidence relating to the availability of training, expertise, equipment, products and support services aids interpretation of the study results and allows consideration of the intervention's applicability within other settings or contexts. Evaluating the implementation or feasibility of a pilot intervention provides evidence which enables judgements relating to the need for and extent of adaptations or modifications on an individual or local systems level (e.g. for each specific ward) [6]. It is only by collecting and reporting these contextual factors that the translation of the research into clinical settings can be facilitated [16].

### Current Practice

Through our extensive preclinical work we found that staff in hospital based stroke care settings experience limited access to training opportunities, OHC policies and in some cases even basic OHC equipment such as toothbrushes and toothpaste [17]. Nursing staff are clearly motivated but general support for the provision of OHC is often inadequate [18]. With a range of competing clinical priorities OHC is often delegated to unregistered members of the stroke multidisciplinary team (MDT) such as clinical support workers, student nurses or even family members [18]. Clearly there is an urgent need to generate evidence to underpin the provision of high quality, well supported OHC in stroke care settings using a pragmatic randomised controlled trial design which compares usual OHC with an experimental complex OHC intervention.

### OHC Intervention

We believe that a successful OHC intervention within the stroke care setting (or system) requires an intervention that is cognisant of the local (and wider healthcare) context and is thus delivered across three levels of care - patients, staff and services. Participant recruitment needs to be inclusive of patients that are most reliant upon staff for their OHC such as those with reduced levels of consciousness, severe cognitive impairment or severe communication impairment

The quality of the OHC delivered is also thought to be dependent on staff knowledge of and attitudes towards OHC [9]. Specially trained staff might be expected to conduct an assessment of OHC, establish the degree and frequency of OHC support required, refer to dental specialists (as required) and develop a care plan in response to patients' OHC needs. In order to support OHC activities OHC tools, products, equipment, specialist training and dental services should be available to both staff and patients on the ward. Some individualisation of the OHC routine by patients and staff might be expected. Staff develop individualised care plans which reflect individual patient's needs and personal preferences based on their level of consciousness, cognitive or physical ability, sensory impairment, rehabilitation targets and pre- and post-stroke oral health. Where possible, patients might be expected to undertake their own OHC.

The success of any planned intervention within the context of a multidisciplinary stroke rehabilitation environment should also consider contributions to the intervention from others within the rehabilitation team including nurses, clinical support workers, physicians and occupational therapists. In addition, specialist dental support from outside the ward (e.g. community dentists, NHS Primary Care dental services) may also be required for more urgent oral health issues.

### Outcomes

Given this degree of complexity identifying a single primary outcome measure to capture the impact of an OHC intervention is problematic [6]. We would anticipate that a multifaceted OHC intervention would impact upon a range of components including for example dental referrals, staff knowledge and patients' oral health. Capturing the impact on dental health alone would need to accommodate a range of dental profiles including those with dentures, natural teeth, a combination of dentures and natural teeth or those who are edentulous (i.e. with neither). Different types of dentition (if any) could be positioned in different locations in each individual's mouth. Thus a carefully selected range of measures may best capture the impact of the intervention across the different dimensions targeted [6].

### Piloting the Implementation

The challenges faced in evaluating such a complex intervention are considerable. Neither quantitative nor qualitative approaches alone would provide an adequate insight into the implementation of the intervention across all three levels of care, from the perspective of all involved and capture the information needed in relation to both effectiveness and feasibility issues. Following our pilot study we also wanted to be able to further refine the planned outcome measures for use within a randomised trial, examine the value of each measure used and to consider the need for any additional measures to capture unexpected effects which became apparent during the course of the pilot.

We aimed to pilot the implementation of a complex OHC intervention across multiple levels of care, adopting an inclusive approach to patient recruitment and a pragmatic approach to the delivery of the experimental OHC intervention. To ensure the data we collected provided a complete picture of the implementation of the complex intervention we used a mixed methods approach, with quantitative and qualitative approaches providing complementary evidence [19]. We aimed to capture evidence relating to the impact of the intervention across components of care, the feasibility of our proposed approach within the pilot site and to highlight any aspects of the intervention that needed to be improved all of which would inform the design and conduct of a future randomised controlled trial.

## Methods

**Figure 1:**
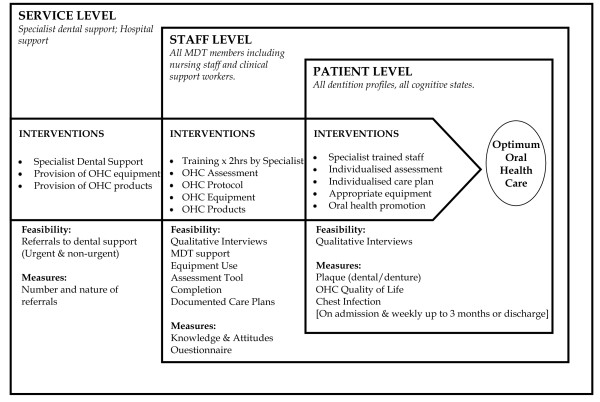
**SOCLE Pilot Study Overview of OHC Complex Intervention**.

We piloted the implementation of a complex experimental OHC intervention in a single stroke care site. The ward was a mixed ward that accepted admissions for acute stroke care and stroke rehabilitation. The intervention included components that targeted three systems of care - (i) patient, (ii) staff and (iii) services. (See Figure 1 for a diagrammatic representation of the complex OHC intervention that was piloted).

### (i) Patients - OHC, Individualised Assessment and Care Plan

Consecutive admissions to a mixed stroke ward (acute or rehabilitation patients) with a primary diagnosis of stroke over a 15 week period were approached to participate in the study. Adopting a pragmatic approach, dentition profile, age, communication ability and cognitive status were not exclusion criteria. In accordance with approval from the Scotland A and the local NHS Lanarkshire Research Ethics Committees, consent was sought from patients to access their medical notes and to measure their dental health and wellbeing. Patients with a mild-moderate degree of language impairment (aphasia) were provided with an adapted version of the information sheet and consent form. Where participants were deemed to be incapacitated because of consciousness levels, severe cognitive or communicative impairment, we approached patients' welfare guardians or carers for consent.

Patients' oral health was expected to be assessed, together with their ability to independently care for their oral health, following admission to the ward. Each patient then had an individualised OHC plan developed for them by the ward staff. In addition, patients then received supported oral health care, had access to the appropriate OHC equipment and products and OHC promotion advice (if required). Thus patients' OHC was better supported than usual care [17].

### (ii) Staff - Training, Tools, Equipment and Specialist Support

From our previous work [17,20] we were aware that staff in stroke care settings lacked access to suitable OHC assessment and protocol tools. Working with an expert Advisory Group (AG) comprised of patient and carer representatives, specialist stroke nurses, physicians, speech and language therapists, dieticians, occupational therapists and dentists from across Scotland the research team had reviewed nationally and internationally published OHC assessment and protocol tools for suitability of use within our planned complex intervention [20]. The AG concluded that the available tools were inadequate and so, through an iterative process, we drafted and refined a stroke-specific OHC assessment and protocol. The assessment tool aimed to comprehensively address all aspects of OHC while the protocol aimed to guide the development of an individual plan of care (for example in terms of the frequency of care or use of products for specific oral health conditions) which would also facilitate patients' return to independent OHC. Staff attending the training were introduced to the OHC assessment and protocol tool and given instruction on their use.

Demonstrating one facet of complexity within the implementation of this complex OHC intervention, staff were not only part of the intervention but they were also participants. At the patient level they were responsible for the delivery of components of the OHC intervention to patients while at staff level they were in receipt of elements of the intervention. Following our preclinical work we were aware of the lack of specialist OHC training opportunities for nursing staff in Scotland [17]. Thus to ensure staff providing the OHC to patients were knowledgeable, had good attitudes to OHC, were skilled in the use of OHC equipment and products and were able to provide appropriate OHC health promotional advice, all staff at the pilot site received an OHC training session. The training session was developed and delivered in conjunction with an experienced specialist gerodontologist (PS) and aimed to consolidate staff knowledge, improve attitudes towards and heighten their awareness of OHC issues. The training also reflected the expert AG's recommendations relating to the ideal context and nature of supporting patients' OHC following stroke. We delivered our two hour training package to all nursing staff across eight training sessions.

Using an adapted Oral Health Care Knowledge and Attitudes Questionnaire [8,9] we assessed the knowledge and attitudes of nursing staff immediately before and after this training component. While the training was delivered to all nursing staff (including clinical support workers and students), other members of the multidisciplinary team were also encouraged to attend but did not complete the questionnaire. Other aspects of the intervention at the staff level included ensuring all staff had adequate ward-level access to basic OHC equipment (e.g. toothbrushes, toothpaste, denture bowls) and products and (as a result of the training) that they were familiar with their recommended use. The use of other rehabilitative equipment (such as adapted handles on toothbrushes) or other MDT interventions relating to OHC was also monitored, as were documented referrals to other members of the MDT for support specifically relating to OHC.

### (iii) Services - Specialist Support and Equipment

Access to specialist dental support can be problematic for stroke wards [17]. We defined the local referral paths for patients admitted to the pilot ward requiring specialist dental support and communicated these arrangements to the ward staff. Referral arrangements differed between patients who were registered with a community dentist and those that were not and between patients with different dental needs (for example urgent and non-urgent). We recorded the number and nature of patient referrals from the ward. We also examined the availability of basic OHC equipment and products through the site procurement systems, to staff on the ward.

### Evidence Captured

For the purposes of the pilot we collected information on the patients' profile on admission (stroke, activities of daily living [21], dentition profile and usual dental routine). Patients' oral health was assessed on admission and then on a weekly basis using measures of dental and denture plaque up to three months after admission or until discharge, whichever was earlier. The research assistant was trained in denture and dental plaque ratings by an experienced gerondontologist and a dental hygienist in an adult special care dentistry setting.

### Effectiveness

At each time point, patients' oral health-related quality of life was also measured using the Oral Health Impact Profile (OHIP) [22] and the General Oral Health Assessment Index (GOHAI) [23]. The presence of dental [24] or denture plaque [25] was measured by a member of the research team (BStG) after demonstrating an appropriate level of accuracy in the scoring procedures. Patients' access to basic OHC equipment and the degree of support they required with their OHC during their stay on the ward was recorded. The incidence of chest infection [26] was also monitored throughout their stay on the ward.

### Feasibility

Staff and patient views on the feasibility of implementing the pilot intervention at service, staff and patient levels were established via semi-structured interviews. Interviews were digitally recorded and fully transcribed. (Interview schedules for the patient and staff interviews can be found in the Additional File). An initial coding framework was systematically developed based on a preliminary review of the data. Patterns were identified using the constant comparative method of data analysis.

### Statistical Analyses

Responses to the OHC Knowledge and Attitudes Questionnaire completed by staff before and after the training were analysed using McNemar test. Plaque data (dental and denture 24-25) were summarised over all visits during their hospital stay (week 1 onwards) as change over baseline in best and worst score in any part of the mouth and then compared using a paired t-test. Changes in oral health related quality of life measures [22,23] were analysed using a Wald test for the overall effect of time in a repeated measures model (after checking for a time by visit interaction, and assuming unstructured correlation across visits). There is no adjustment for multiple comparisons, so the reader should be aware that there is a risk of over interpreting these data given the large number of significance tests undertaken.

## Results

The results are presented below as they relate to the three levels at which the complex intervention applied (i) patient, (ii) staff and (iii) service levels. While it may be more conventional to present quantitative and qualitative findings separately, we have chosen to present the quantitative and qualitative findings alongside each other [27] thus demonstrating the manner in which the two approaches contributed to informing the development and design of the next stage of this work.

### (i) Patient - Dental and denture plaque measures, Oral Health Quality of life

Over the course of the 15 week recruitment period there were a total of 81 consecutive admissions to the ward with a primary diagnosis of stroke. The local Early Supported Discharge (ESD) service resulted in 23 potentially eligible patients admitted with stroke being discharged before they could be included in the study (within 24-48 hours of admission). A further 15 declined to participate (n = 7 patient; 8 carer) or died (n = 3) (Figure 1). Thus 40 participants were recruited to this study and had a mean age of 72 (SD 12.2) (range 45-92) years, were experiencing a broad range of stroke related impairment (modified Rankin Scale [mRS]) and presented with a range of dental profiles (Table 1).

**Table 1:** Participants

Participants Profile	Frequency (n = 40)	Percentage
Male	23	57.5
Female	17	42.5

Admitted from		
Home	35	87.5
Care home	2	5
Elsewhere in Hospital	3	7.5

Impaired Dominant Hand/Arm		
Yes	15	37.5
No	25	62.5

Communication		
Normal	16	40
Aphasia	13	32.5
Dysarthria	6	15
None*	5	12.5

Consciousness		
Alert	31	77.5
Confused/Disorientated	6	15
Reduced Consciousness	1	2.5
Unconscious	2	5

Infarct/Haemorrhage		
Infarct	37	92.5
Haemorrhage	3	7.5

Site of Lesion		
Right	17	42.5
Left	22	55
Unclear	1	2.5
TACI	8	20
PACI	9	22.5
LACI	11	27.5
Other	12	30

Impairment (modified Rankin Scale)		
0-2 Slight to no disability	16	40
3-5 Moderate or severe disability	23	60

Dentition**		
Natural Teeth	14	35
Dentures	29 (full 23; partial 6)	72.5
Edentulous	2	5

Location of Dentures	(n = 29)	
Hospital	24	82.8
At home	3	10.3
Missing	4	13.8

Dentures Worn	(n = 29)	
Yes (4 only occasionally)	18	45
No	11	27.5

Key: On admission to the study most participants were in the very acute stages following stroke onset (1 day of stroke onset) with the maximum of 22 days after stroke (top three longest post onset on admission were 8-22 days and were admissions from elsewhere across the hospital). Age range was 45-92 years of age. * = (cognitive or decreased consciousness). **Some patients had both natural teeth and partial dentures. TACI = Total Anterior Circulation Infarct: PACI = Partial Anterior Circulation Infarct: LACI = Lacunar Infarct

**Figure 2:**
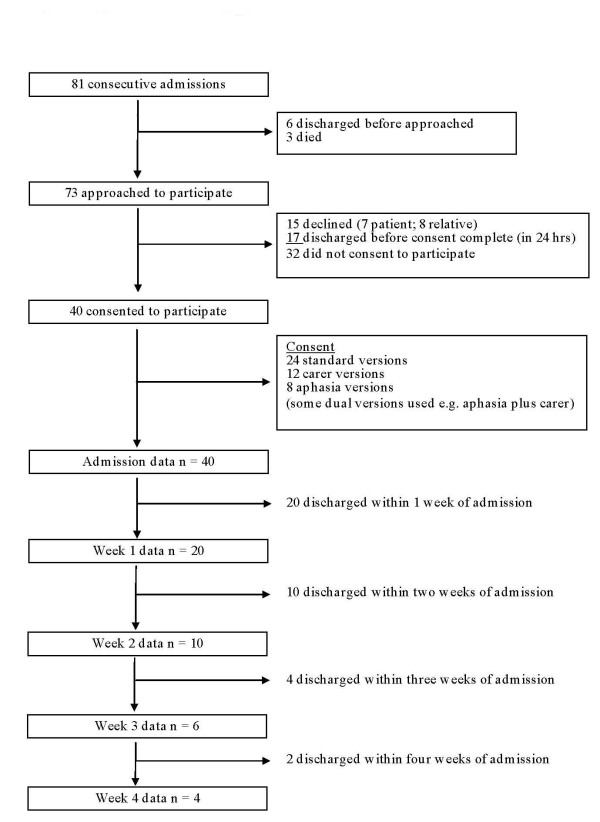
**Recruitment Flowchart:Overview of patients recruitment to and discharge from the study**.

Communication impairments were common with almost half experiencing aphasia or dysarthria while an additional five participants were unable to communicate because of cognitive impairment or decreased consciousness. Consequently, we approached 12 carers for consent and used accessible versions of the consent tools with eight people who had aphasia (4 were consented using dual versions of the tools). Patient consent was not required before staff could assess or care for the patient's oral health. Consent related to permission for the research team to access the individual patient's healthcare notes, to measure the patient's oral health, oral health related quality of life and (where appropriate) to obtain their views relating to the OHC received in the ward. Chest infection [26] was rare with only one participant found to have indications of a chest infection on recruitment to the study. We collected baseline admission data from all 40 participants, of which, only 20 remained on the ward one week later. Numbers of patients remaining in hospital continued to decrease over the following weeks (Figure 2).

#### Effectiveness Data

The agreement and accuracy for the outcome assessment for both the detail and denture plaque was excellent. For both outcomes two raters analysed the same subjects. The kappa statistic for the dental plaque inter-rater agreement is 0.90 (95% confidence interval 0.83 to 0.97), with a P-value for the test of zero kappa being P = 1.16 * 10^-42 ^and the corresponding kappa statistic for the denture plaque is 0.95 (95% confidence interval 0.84 to 1.00), with a P-value for test of zero kappa being P = 1.44 * 10^-14 ^(Additional File 1, Table S1).

The cleanliness of participants' teeth was measured on admission and then on a weekly basis thereafter using a dental plaque score [24]. A five point scale was used to grade the presence of plaque on six different regions of the teeth (six sextants). For the purposes of analysis and to accommodate the participants' rapid discharge, we compared patients' baseline scores with their best and worst scores at any time throughout their hospital stay (weeks 2-5). As teeth in any mouth are only as clean as the tooth with the most plaque, we compared the sextant with the most plaque on admission with the sextant with the most plaque at later assessment points and the sextant with the least plaque on admission with the sextant with the least plaque at later assessment points. There was no evidence of a significant difference in worst plaque rating (p = 0.4) or best plaque rating (p = 0.081; Table 2).

**Table 2:** Denture and Dental Plaque Scores

Type of plaque	Baseline			After			After - Baseline			
**Patients with Dentures**	N	Mean	SD	N	Mean	SD	N	Mean	SD	P-value
Denture Plaque										
Minimum (Best) Score	23	0.70	0.79	9	0.22	0.44	9	-0.56	0.53	0.013*
Mean Score	23	0.98	0.69	9	0.75	0.48	9	-0.22	0.67	0.35
Maximum (Worst) Score	23	1.26	0.81	9	1.11	0.78	9	-0.31	0.59	0.16
										

**Patients with Natural Teeth**										
Dental Plaque										
Minimum (Best) Score	14	1.29	0.73	10	0.90	0.74	10	-0.30	0.48	0.081
Mean Score	14	1.57	0.80	10	1.32	0.80	10	-0.20	0.42	0.17
Maximum (Worst) Score	14	1.86	0.86	10	1.80	1.03	10	-0.10	0.37	0.40

**Denture and Dental Plaque Scores **-Reflects the 23 participants that wore dentures while in hospital and 14 participants with natural teeth. In some cases a single individual could have both denture and dental plaque scores. Two individuals were edentulous and are not represented within this table.

Denture Score is on a scale of 0-4 (0 = none [0%], 1 = light [1-25%], 2 = moderate [26-50%], 3 = heavy [51-75%], 4 = very heavy [76-100%], whereas Dental Score is on a scale 0-3 [0 = no debris, 1 = soft debris <33%, 2 = soft debris 34-67%, 3 = soft debris 68-100%]. The data shown is calculated across all sections (Dentures - six + fitting plate; Dental - five) at baseline and then 'After' (across all of weeks 2,3,4 or 5). The P-value is for a one sample 2-sided paired t-test on whether the difference After-Before is significantly different from zero. * = significant at p < 0.05.

Similarly, participants' dentures were examined for plaque during their stay in hospital using a denture plaque score [25]. Plaque was rated on a four point scale across seven different denture regions (six sextants and the fitting surface). As above, comparisons of the best and worst plaque scores on recruitment were made with plaque scores across all data collection points. There was no evidence of a significant difference in worst plaque rating (p = 0.16) but there was some indication that the cleanest parts of the patients' mouths became even cleaner during their stay in the stroke ward (p = 0.013; Table 2).

The dental and denture plaque efficacy results must however be interpreted with great caution because of the high attrition rate observed amongst the participants. Furthermore, in the before-after comparison, the 'after' variable is an average of all follow-up weeks (2, 3, 4 or 5) to enable the sparse data to be included. The estimate of the difference observed in plaque scores, while apparently statistically significant, is therefore not robust.

During this pilot we measured oral health related quality of life (GOHAI and the OHIP) and compared changes from the point of study recruitment over time. Using the GOHAI participants reported increased limitations in the kinds or amounts of food eaten (p = 0.035), greater trouble biting or chewing (p = 0.041) and more sensitive teeth or gums (p = 0.043) during their stay in the ward (Additional File 1: Table S2). In contrast, on the OHIP participants described fewer difficulties speaking (p = 0.035), less self consciousness (p = 0.031) and tension about their teeth (p = 0.0057) during their stay. Similarly, they had fewer problems relaxing (p = 0.02), with life satisfaction (p = 0.0042) and or functioning (p = 0.064) over time (Additional File 1: Table S3).

#### Feasibility - Patients' views

We collected qualitative data relating to implementation from 15 of the participants using semi-structured interviews (details for not interviewing the remaining participants can be found in Additional File 1: Table S4). Although some patients found it difficult to recall the provision of OHC, others recalled difficulty conducting OHC. Some participants believed that OHC was not a priority for staff who were perceived to be more focused on clinical signs such as blood pressure.

That [OHC] was put to the back, everything else came first... Actually, I had to say to them "Can I have my teeth, something for my teeth?" you know. Patient Identification (ID): 12

Others described how they lacked interest in their own OHC after their stroke or that OHC had been problematic because of missing equipment.

Didn't attend to them a great deal. Patient ID:1

I didn't take anything in with me so I didn't really do anything with them (dentures) when I was in over the three days. Patient ID:8

On the ward, I didn't seem to get was a dish to put my teeth out at night to sort of settle down, probably because there wasn't a dish available .....I had one for about two nights and then it disappeared. Patient ID:2

*P: No. I couldn't because I didn't bring anything [OHC equipment] with me*.

Researcher (R): Did you ask for anything?

*P: No ... I didn't know whether I could or not*.

R: So how long were you without?

P: The first three days I was in .... Patient ID:8

In contrast, other patients recalled a keen awareness of OHC issues, well supported OHC (in the main provided by nursing staff) and the provision of basic OHC equipment, if required.

I brought my toothbrush but em ... I forgot my toothpaste but they gave me toothpaste no bother. Patient ID:15

Oh yes if I was needing anything I could get it, aye. Patient ID:6

I told them I could do it myself no problem. Patient ID:6

So much time in hand...just sitting there running the tongue over the teeth and going "Oh ho! I think a wee brush is in order here". Patient ID:62

Support in adapting their OHC routine to overcome stroke related impairment(s) was described by some patients but others appeared unaware this sort of support had been available.

They shows you what to do. Patient ID:11

R Does it [your upper limb weakness] affect you looking after you teeth at all?

P No, no. 'Cause I just take them [dentures] out and hold them in my hand. Patient ID:62

I didn't know that they could help me with that as well. Patient ID:8

Well, if they would have suggested that I try, I would have. ... I never felt fit or able to do it. Patient ID:63

I mean I don't see it as a big deal as to who cleans them. You know, as long as they're cleaned. ....There is always someone there if I say to them "Do you mind giving my teeth a brush?". Patient ID:42

### (ii) Staff - Training, Tools, OHC Equipment and Products and Support

A total of 26 nursing staff (18 trained nurses, 5 clinical support workers and 3 nursing students) participated in the study. We also attempted to include all members of the multidisciplinary team.

#### Effectiveness Data

The numbers of nursing staff completing the adapted Knowledge and Skills Questionnaire [8,9] immediately before and after the training were small (n = 23 and n = 22 respectively). In addition, levels of OHC knowledge were in some areas excellent and so analyses were hindered by the ceiling effect of maximum scores from the outset (Q5 & 6; Additional File 1: Table S5). However, where significant changes were observed, most demonstrated significantly improved attitudes and knowledge following the training. Additional File 1: Table S5 reports results that were significant (P < 0.05) or approached significance (P > 0.05 but P < 0.07). Responses to two questions however failed to demonstrate a unidirectional shift (Q10 & 21) highlighting some OHC aspects that require more specific instruction within the training session.

Staff were introduced to the OHC assessment and protocol during the training sessions and were encouraged to use them to establish individual patients' ability to conduct independent oral care and to inform individualised OHC plans. Rehabilitation issues were emphasised as was ensuring patients' good oral health. We found that in some cases acceptance of our expert Advisory Group pilot OHC protocol was not straightforward. For example, oral foam sponges were favoured by many staff who found them helpful in caring for people's oral health and wanted to continue to use them. Other staff had concerns about using them which echoed the concerns of our Advisory Group. In exploring this issue within the pilot site we identified a local NHS Hazard Notice which stated that oral foam sponges 'should not be soaked in water before use'. As a result they continued to be used during this pilot by some members of staff but within the recommendations of the Hazard Notice. Similarly, some staff were concerned about our recommendation to use a gauze covered gloved finger for some OHC as they perceived this to be in conflict with a local health and safety guideline. Upon investigation we found no health and safety directives preventing staff from using this method to support OHC and fed this back to the staff. Although staff had access to a denture marking kit on the ward, and despite the clear direction within the protocol that patients' dentures should be named on admission, adherence to this recommendation was poor. Of 18 participants with dentures admitted to the ward following a stroke, only six were named.

...they just said that they didn't want to have the teeth, the name put on their teeth. That did cause a, not all families, but a couple of families we had and they just said "No". They didn't want their name put on their teeth. Staff ID: 7

These issues reflect the challenges of communicating and implementing a complex intervention which required staff to apply a high number of behaviours. At the same time, (as described by the MRC guidance on the evaluation of complex interventions [6]) it is important to retain some flexibility, allowing adaptation of the intervention to the local context and ensuring the intervention can be tailored for individual OHC needs.

All members of the MDT were invited to the specialist training sessions. Two occupational therapists, two physiotherapy staff, one speech and language therapist and a speech and language therapy student attended. Some members of the local community Primary Care Dental Services also attended. Despite this, nursing staff still perceived they had little or no input from the rest of the MDT. We found no record of a referral to the MDT specifically for OHC related support, nor were any adaptive tools or equipment (for example adapted handles for toothbrushes) issued to support participants' resumption of independent OHC during the pilot.

R Did you find you got more input from the other members of the team? From the OT? From the speech and language therapist? From the physio in relation to their oral health?

S (No) No ((shakes head))...The speech and language yeah. The speech and language normally come to us and tell us if there is anything, kinda thing but ...nope

R But the OT? Like adapted handles on toothbrushes or how best to position...?

S ((Shakes head)) Sorry, If you want the truth, nope. Staff ID: 4

#### Feasibility - Staff Views

During the qualitative interview component to explore feasibility issues, the staff described the guidance within the OHC protocol relating to the care and cleaning of dentures as particularly helpful. The dental training was perceived not only to have had positive benefits for the care patients received, but a number of staff also commented on how the oral care practices in their own homes had changed as a result too.

.. its helping me at home ((laughs)) cause I'm like that, "You don't have to buy that you know", "Brush your teeth", "Brush your tongue", and this kinda thing...and even the keeping the toothpaste in your mouth for so long and ((laughs)) ...I've got my whole family saying "Don't rinse your mouth out straight away". Staff ID: 4

The recommendation to use soap and water for cleaning dentures was clearly novel to most members of staff and the introduction to other OHC products within the training session was found to be very useful in cases of dry mouth and staphylococcal mucositis. In addition, staff described the availability of basic OHC equipment on the ward as essential, as reliance on family members for these items was felt to be problematic.

...not always the denture bowl. They don't often think of the denture bowl. But that's fine. We do have a good supply of denture bowls so we don't mind that in the least. Staff ID: 4

Staff reported they liked the OHC assessment and protocol, in particular commenting on the relevance of their content and the ease of use. The fact that all members of nursing staff (nurses and clinical support workers) were equally able to use the tool was looked upon favourably.

There was a lot of information that let you deal with people's oral health that have had a stroke.... Like a lot of things that you probably maybe wouldn't think of in your own mind to check. This [OHC assessment] allowed you to check for things, that's what I liked about it. Staff ID: 7

...nothing irrelevant and nothing that you couldn't really do at the patients' bedside. Nothing that you had to go and trawl through the notes or ask a nurse who had access to lab results or go to ask a doctor...everything thing was there you either got from the patients' notes or the patients would give you the information themselves and actually looking at their mouths as well. Staff ID: 2

Everybody that we managed to get onto the training. So everybody was able to do the assessment tools. And I mean, who, who would actually do it? I mean. I would just have to make a ball park guess. I would say kinda fifty-fifty. Staff ID: 2

For this pilot study we deliberately adopted a pragmatic approach to completion of the assessment tool on the ward, allowing tailoring of the intervention to individual patient's needs and local integration of the tool into ward routine. Senior members of staff on the ward felt tool completion was acceptable and sufficient to inform their clinical practice. As this was a pilot study we further explored the factors which may (from the point of view of ward staff) have impacted upon completion of the assessment or use of the protocol including; difficulty eliciting information from some patients; recruitment of patients with a diagnosis of stroke from mixed admissions to the ward; sharing of the assessment task over shifts; the standard completion rate and the perception of the research activities as tasks to be completed by others.

It was a very good tool but like any tool it's as good as the person that was actually using it. Staff ID: 2

### (iii) Services - Specialist Support, Management Support and Equipment

#### Effectiveness and Feasibility Data

During the pilot study no patient required an urgent or a non-urgent referral to a dental specialist. We found that less than half of the patients in the study reported they were registered with a dentist (n = 16; 40%) and only a quarter (n = 11; 28%) were able to provide their dentist's name or other contact details. This finding has implications for future studies in anticipating the type of specialist service level support for patients in a large randomised trial. Although no patients required treatment from a specialist dentist other service links, in particular an on-site denture repair laboratory (unknown to the staff prior to this study), were used to patients' benefit.

...there wasn't really a need for onwards referral. It seemed to be that people's mouths seemed to be fairly good when they came in. Staff ID: 2

*So a couple of weeks ago a wee lady dropped her false teeth and they cracked in half. So the one thing that came from that was, I got them sent down, they were fixed that day and they were back up again. Whereas a few months ago, we wouldn't have known [about the onsite denture repair lab], we'd have been giving them to the family to take to the community dentist*.

 Staff ID: 2

We found that most basic OHC equipment was available to staff within the pilot site through the usual hospital procurement and supplies system. A denture naming kit (for naming of all dentures upon admission to the ward as recommended by our expert Advisory Group) was unavailable and sourcing a commercial denture naming kit proved impossible. The local Lanarkshire Primary Care Dental Services provided one of their own kits. Some dental care products (e.g. a saliva replacement gel) were also unavailable and were included on the hospital procurement list during the study.

## Discussion

### Summary of study

The main outcome of this study was the successful development and implementation of a highly complex OHC intervention. The benefits experienced at patient level relied upon the active cascade of various components of the intervention through service, staff and patient levels of care. Service support impacted upon staff, and staff support in turn impacted upon nurses' and clinical support workers' ability to care for or assist patients to conduct independent OHC, all of which impacted upon patients' oral health.

Our mixed methods approach to evaluating the implementation of the complex intervention provided important complete and complementary evidence and guidance. This information has been invaluable in establishing the nature of the OHC intervention delivery, informing subsequent refinements and our plans to design an evaluation of the effectiveness of the intervention using a randomised controlled trial design.

### Mixed Methods Approach

By collecting both quantitative and qualitative data we were able to identify not only the comprehensive and complementary evidence relating to the implementation of a complex intervention that comprised of many components, but we captured information relating to the interactions (anticipated and unanticipated) between those components. For example, although we had adopted an inclusive approach to the recruitment of patients following stroke it was clear that staff believed that implementation of the OHC protocol might have been more consistent if recruitment occurred across the mixed ward care setting rather than based only upon the reason for admission. While training both qualified nursing staff and clinical support workers from the pilot ward was seen as very important, some aspects of the intervention were not well received (marking names on dentures, using gauze covered fingers and suspending the use of foam swabs). Further evidence to support the use of these approaches will be built into the training sessions and addressed from the outset in further sites. Our mixed methods approach provided a wealth of information on the feasibility of the pilot intervention in relation to the methodological issues such as exact specification of our intervention and the planned outcomes. However, as with most pilot studies, our study was based on a small number of patients and staff from a single clinical site, and although the data generated were of high quality, there was inevitably limited insight into the more process orientated uncertainties such as recruitment rates for a multicentre, definitive trial.

Given the limited availability of similar work within this topic area [7] it is difficult to draw comparisons with previous work. Some OHC trials delivered to people resident within a nursing home setting did successfully impact upon staff knowledge and attitudes [8,9] but the patients, staff and setting were different from those in this current project [8,9,13]. Unlike previous studies in the field [8-13], the inclusive approach to patient recruitment and recognition of the interconnection between the three dimensions of service, staff and patient levels of care was shown to be feasible.

### Implications for future research

Our approach to the definition, description and implementation of a highly complex OHC intervention delivered within a stroke care setting was feasible and facilitated the delivery and measurement of the impact of the pilot intervention. The use of a mixed methods approach to evaluate the implementation of the intervention also proved feasible. We now plan to establish the effectiveness of the experimental OHC intervention within a clinical trial design. Though there may be considerable resource implications in continuing to use a mixed methods approach within a randomised controlled trial design [28], the complementary nature of the data collected and the resulting completeness of the evidence relating to the operationalisation of the OHC intervention 'in context' across different clinical settings and systems, would be highly informative. Such information would facilitate translation of any emerging evidence into clinical practice by providing insights into the interaction of the intervention components within different contexts. Thus the feasibility of adopting a mixed methods approach within a trial design is something that we plan to explore.

The study reported here was conducted in a single pilot site with staff receiving intensive support from a dedicated research nurse. We anticipate some challenges rolling out the intervention and data collection procedures across numerous sites with less intensive research support. Other possible approaches will be explored, such as providing specialist training to an 'OHC champion' who in turn would be expected to deliver the training to smaller ward based groups as available. An alternative approach might be to explore the possibility of developing an independent web-based learning tool in relation to OHC. Where possible, for data collection purposes, we would seek to ensure the trial received the support of the UK Stroke Research Network.

In our pilot we established the rate of admission, retention and length of patient stay within the ward, the possible requirement for specialist dental services, the frequency of patients' registration with community dentists and the need to monitor access to community dentists following discharge. Time from admission to discharge after stroke was much shorter than initially anticipated. A relatively new local Early Supported Discharge team and evidence to support the effectiveness of community based rehabilitation [29] resulted in half of the patients with stroke being discharged within a week of their admission. The high rate of attrition from initial recruitment numbers upon admission is reflective of the current NHS in-patient stroke management process.

Our planned evaluation of the effectiveness of the delivery of this OHC intervention is supported by the success of this pilot study. We would anticipate that the contextual components to our intervention will ultimately facilitate the translation of the work into clinical practice. The quantitative data collected within the study will also facilitate sample size calculations, which are being developed for a stepped wedge cluster design [30,31]. Separate sample size calculations will be based on patient and staff measures with the appropriate number of sites recruited to ensure an adequate sample size is achieved.

Although multiple primary outcomes may be more acceptable within such a complex evaluation we still need to define those that are of primary importance and those that are less so. The incidence of chest infection within the pilot site was very low during the study (a single individual). Local anecdotal evidence and a recent trial [12] may indicate that changes in the incidence of this serious complication may be positively impacted upon by the conduct of good OHC. The impact of a patient's oral health upon their quality of life remains an important measure, with measures of dental and denture plaque robust indicators of how clean the mouth is.

From the service level, impacts on the patients' systemic health are likely to impact upon length of hospital stay but delivery of the specialist training and ensuring access to OHC equipment and products incurs some additional costs. Inclusion of an economic component in a future evaluation will be essential. Use of specialist services will be a key future outcome looking at onward referral rates to community dentists. We will also explore the possibility of monitoring attendance at community dentists after discharge through NHS data linkage investigation. During the course of our semi-structured interviews with patients, several mentioned a recent or pending dental appointment and our planned future work will aim to capture this possible oral health promotion impact too.

## Conclusions

Our use of a mixed methods approach to our pilot study facilitated an extensive comprehensive insight into the implementation of a complex OHC intervention across a number of levels of care in a single stroke ward. The findings of the study demonstrated the feasibility (and areas for further refinement) of the recruitment and consent processes, intervention and outcome measures. Such a multi-dimensional, pragmatic, complete, mixed methods approach to the development of the intervention and trial will ensure the planned trial will address some of the methodological weaknesses of previously conducted work in this topic area. The insights and understanding gathered through this important implementation work will inform the design and development of a trial to evaluate the effectiveness of the experimental OHC intervention across stroke care settings and (should it prove effective) facilitate the translation of the evidence into clinical settings.

## List of abbreviations

AG: Advisory Group; ESD: Early Supported Discharge; GOHAI: General Oral Health Assessment Index; LACI: Lacunar Infarct; MDT: multidisciplinary team; mRS: Modified Rankin Scale; MRC: Medical Research Council; OHC: Oral health care; OHIP: Oral Health Impact Profile; PACI: Partial Anterior Circulation Infarct; RCT: Randomised Controlled Trial; SD: Standard Deviation. TACI: Total Anterior Circulation Infarct.

Key to Quotations: P: patient; S: staff; R: researcher; ID: identification number; (...): single bracket indicates speech unclear but possible interpretation offered; ((...)): double bracket indicates an action that occurs e.g. ((laughter)); [...]: explanation of text.

## Competing interests

The authors declare that they have no competing interests.

## Authors' contributions

MB Developed the intervention, designed, conducted and led the project, participated in data collection and quantitative analysis, conducted the qualitative analysis and drafting the manuscript. DS Participated in the design and coordination of the study, analysis and interpretation of the data and helped to revise the manuscript. JN Participated in the design and coordination of the study, conducted the statistical analysis and helped to revise the manuscript. PL Participated in the design and coordination of the study, analysis and interpretation of the data and helped to revise the manuscript. CC Participated in the design and coordination of the study, analysis and interpretation of the data and helped to revise the manuscript. BStG Consented and recruited participants, collected quantitative and qualitative data, contributed to the analysis and interpretation of the data and helped to revise the manuscript. PS Provided specialist input to the intervention, training component, outcome measurement and analysis. All authors have read and approved the final manuscript.

## Supplementary Material

Additional file 1 **Supplementary Tables and Interview Schedules**. Data on intra-rater dental and denture plaque ratings; General Oral Health Assessment Index; Oral Health Impact Profile; Interview participants; knowledge and attitudes Questionnaire; Interview schedule for participants (patients and staff). Click here for file
