# Supplementary material for: Developing and evaluating the implementation of a complex intervention: using mixed methods to inform the design of a randomised controlled trial of an oral healthcare intervention after stroke
Source: Trials. 2011 Jul 5;12:168. doi: 10.1186/1745-6215-12-168 (PMC3155479; doi:10.1186/1745-6215-12-168)
Supplement: Additional file 1 — Supplementary Tables and Interview Schedules. Data on intra-rater dental and denture plaque ratings; General Oral Health Assessment Index; Oral Health Impact Profile; Interview participants; knowledge and attitudes Questionnaire; Interview schedule for participants (patients and staff). [file 1745-6215-12-168-S1.DOC]

**Table S1**

1. Dental Plaque Training - Inter-rater reliability

| **Dental Plaque** | **Rater 2** |  |  |
| --- | --- | --- | --- |
|  | **< 1/3** | **1/3 – 2/3** | **>2/3** |
| **Rater 1 <1/3** | 38 | 1 | 0 |
| **1/3-2/3** | 3 | 55 | 1 |
| **>2/3** | 0 | 3 | 24 |

Dental plaque is scored on a scale of 0-3 [0=no debris, 1=soft debris <33%, 2= soft debris 34-67%, 3=soft debris 68-100%].

(b) Denture Plaque Training – Inter-rater reliability

| **Denture plaque** | **Rater 2** |  |  |  |  |
| --- | --- | --- | --- | --- | --- |
|  | **None** | **Light** | **Moderate** | **Heavy** | **Very Heavy** |
| **Rater 1 None** | 6 | 0 | 0 | 0 | 0 |
| **Light** | 0 | 5 | 0 | 0 | 0 |
| **Moderate** | 0 | 1 | 4 | 0 | 0 |
| **Heavy** | 0 | 0 | 0 | 6 | 0 |
| **Very heavy** | 0 | 0 | 0 | 0 | 2 |

Denture plaque is scored on a scale of 0-4 (0=none [0%], 1 = light [1-25%], 2=moderate [26-50%], 3=heavy [51-75%], 4=very heavy [76-100%].

**Table S2 – General Oral Health Assessment Index**

| **Question** | **Baseline** | | | **Time 1** | | | **Time 2** | | | **P-value** |
| --- | --- | --- | --- | --- | --- | --- | --- | --- | --- | --- |
|  | N | Mean | SD | N | Mean | SD | N | Mean | SD |  |
| Q2. Did you limit the kinds or amounts of food you eat because of problems with your teeth or dentures? | 32 | 2.03 | 1.33 | 15 | 1.80 | 1.32 | 8 | 3.52 | 1.58 | 0.035* |
| Q3. Did you have trouble biting or chewing any kinds of foods, such as firm meat or apples? | 32 | 2.13 | 1.39 | 15 | 2.47 | 1.60 | 8 | 3.38 | 1.41 | 0.041* |
| Q4. Were you able to swallow comfortably? | 34 | 4.26 | 1.19 | 15 | 4.33 | 1.05 | 8 | 3.73 | 1.34 | 0.24 |
| Q5. Have your teeth or dentures prevented you from speaking the way you wanted? | 32 | 1.97 | 1.28 | 15 | 1.47 | 1.13 | 8 | 1.81 | 1.10 | 0.38 |
| Q6. Were you able to eat anything without feeling discomfort? | 33 | 4.06 | 1.34 | 15 | 3.53 | 1.64 | 8 | 3.79 | 1.53 | 0.41 |
| Q7. Did you limit contacts with people because of the condition of your teeth or dentures? | 33 | 1.33 | 0.89 | 15 | 1.13 | 0.52 | 8 | 1.81 | 1.46 | 0.26 |
| Q8. Were you pleased or happy with the looks of your teeth and gums, or dentures? | 32 | 3.97 | 1.51 | 15 | 3.73 | 1.33 | 8 | 3.71 | 1.46 | 0.79 |
| Q9. Did you use medication to relieve pain or discomfort from around your mouth? | 33 | 1.06 | 0.35 | 15 | 1.00 | 0.00 | 8 | 1.00 | 0.00 | 0.60 |
| Q10. Were you worried or concerned about the problems with your teeth, gums or dentures? | 33 | 1.30 | 0.81 | 15 | 1.40 | 1.12 | 8 | 1.46 | 0.85 | 0.78 |
| Q11. Did you feel nervous of self-conscious because of problems with your teeth, gums or dentures? | 33 | 1.58 | 1.06 | 15 | 1.53 | 1.13 | 8 | 1.69 | 0.76 | 0.89 |
| Q12. Did you feel uncomfortable eating in front of people because of problems with your teeth or dentures? | 32 | 1.63 | 1.04 | 15 | 1.33 | 0.72 | 8 | 1.33 | 0.47 | 0.42 |
| Q13. Were your teeth or gums sensitive to hot, cold or sweets? | 32 | 1.66 | 1.15 | 15 | 1.33 | 0.90 | 8 | 1.83 | 1.46 | 0.043* |

**General Oral Health Assessment Index** - questions 2-13 on 5 point scale (1=never, 2=seldom, 3=sometimes, 4=often, 5=always). N, mean, SD at baseline, 1 week (time 1, ~15 responses) and time 2 (the average of available data from weeks 2, 3, 4, and 5: in general there were 7 responses at week 2, and 4, 1 and 1 at weeks 3, 4 and 5). The P-value refers to a Wald test for the overall effect of time (3 periods – baseline, time 1 and time 2) in a repeated measures ANOVA with an assumed full covariance structure. * = p < 0.05.

**Table S3 – Oral Health Impact Profile**

| **Question** | **Baseline** | | | **Time 1** | | | **Time 2** | | | **P-value** |
| --- | --- | --- | --- | --- | --- | --- | --- | --- | --- | --- |
|  | N | Mean | SD | N | Mean | SD | N | Mean | SD |  |
| Q1. Have you had trouble pronouncing any words because of problems with your teeth, mouth or dentures? | 34 | 3.79 | 1.51 | 15 | 4.73 | 1.03 | 8 | 3.98 | 1.20 | 0.035* |
| Q2. Have you felt that your sense of taste has worsened because of problems with your teeth, mouth or dentures? | 30 | 4.53 | 1.11 | 15 | 4.93 | 0.26 | 8 | 4.50 | 1.07 | 0.10 |
| Q3. Have you had painful aching in your mouth? | 34 | 4.79 | 0.64 | 15 | 4.80 | 0.77 | 8 | 5.00 | 0.00 | 0.093 |
| Q4. Have you found it uncomfortable to eat any foods because of problems with your teeth, mouth or dentures? | 33 | 4.18 | 1.24 | 15 | 4.53 | 0.74 | 8 | 4.40 | 0.77 | 0.37 |
| Q5. Have you been self conscious because of your teeth, mouth or dentures? | 33 | 3.85 | 1.66 | 15 | 4.67 | 0.90 | 7 | 4.45 | 0.84 | 0.031* |
| Q6. Have you felt tense because of problems with your teeth, mouth or dentures? | 33 | 4.30 | 1.29 | 15 | 4.87 | 0.52 | 7 | 5.00 | 0.00 | 0.0057** |
| Q7. Has your diet been unsatisfactory because of problems with your teeth, mouth or dentures? | 32 | 4.16 | 1.22 | 15 | 4.27 | 0.96 | 8 | 3.83 | 0.79 | 0.46 |
| Q8. Have you had to interrupt meals because of problems with your teeth, mouth or dentures? | 32 | 4.09 | 1.23 | 15 | 4.47 | 0.83 | 8 | 4.38 | 0.92 | 0.32 |
| Q9. Have you found it difficult to relax because of problems with your teeth, mouth or dentures? | 31 | 4.55 | 0.96 | 15 | 5.00 | 0.00 | 8 | 4.88 | 0.35 | 0.020* |
| Q10. have you been a bit embarrassed because of problems with your teeth, mouth or dentures? | 32 | 3.94 | 1.56 | 15 | 4.60 | 0.91 | 8 | 4.25 | 1.39 | 0.10 |
| Q11. Have you been a bit irritable with other people because of problems with your teeth, mouth or dentures? | 33 | 4.79 | 0.65 | 15 | 4.87 | 0.52 | 8 | 5.00 | 0.00 | 0.16 |
| Q12. have you had difficulty doing you usual jobs because of problems with your teeth, mouth or dentures? | 31 | 4.87 | 0.43 | 15 | 5.00 | 0.00 | 8 | 4.94 | 0.18 | 0.13 |
| Q13. Have you felt that life in general was less satisfying because of problems with your teeth, mouth or dentures? | 32 | 4.31 | 1.20 | 15 | 4.87 | 0.52 | 8 | 4.92 | 0.24 | 0.0042** |
| Q14. Have you been totally unable to function because of problems with your teeth, mouth or dentures? | 32 | 4.72 | 0.81 | 15 | 4.87 | 0.52 | 8 | 5.00 | 0.00 | 0.064 |

**Oral Health Impact Profile** - questions 1-14 on 5 point scale (1=very often, 2=fairly often, 3=occasionally, 4=hardly ever, 5=never). Note that response=6 (don’t know) was excluded. N, mean, SD at baseline, 1 week (time 1, ~15 responses) and time 2 (the average of available data from weeks 2, 3, 4, and 5: in general there were 7 responses at week 2, and 4, 1 and 1 at weeks 3, 4 and 5). The P-value refers to a Wald test for the overall effect of time (3 periods – baseline, time 1 and time 2) in a repeated measures ANOVA with an assumed full covariance structure. * = p < 0.05; ** = p < 0.01

**Table S4 Interviewed Participants (n = 15)**

| **Participant** | **Aphasia** | **Length of Stay** | **Location** | **Discharge from Pilot site** |
| --- | --- | --- | --- | --- |
| 1 | No | 1 | H | After |
| 2 | No | 1 | H | Before |
| 6 | Aphasia | 1 | Hm | After |
| 8 | No | <1 | Hm | After |
| 11 | Aphasia | 3 | H | Before |
| 12 | No | <1 | Hm | After |
| 15 | No | <1 | Hm | After |
| 21 | No | <1 | Hm | After |
| 26 | No | <1 | H | Before |
| 32 | No | <1 | H | After |
| 34 | No | 2 | H | Before |
| 42 | No | 1 | H | Before |
| 56 | No | <1 | H | After |
| 62 | No | <1 | Hm | After |
| 63 | No | <1 | Hm | After |

Reasons for no interview data = 6 severe communication impairment; 5 cognitive problems; 4 died; 4 inpatients; 3 interviews not completed before RA contract ended; 1 advised against home visit; 1 unable to contact; 1 geographical ; 1 end of life care.

**Table S5: - Knowledge and Attitude Questionnaire** ^9^

| **Questions 1-25 (True-False)** | | **11 (T-T)** | **12 (T-F)** | **21 (F-T)** | **22 (F-F)** | **McNemar**  **p-value** |
| --- | --- | --- | --- | --- | --- | --- |
| **Q1** | Dentures should be taken out at night | 17(85%) | 0(0%) | 2(10%) | 1(5%) | 0.50 |
| **Q2** | Denture cleaning solutions remove dirt from dentures without need to brush | 0(0%) | 2(10%) | 1(5%) | 18(86%) | 1.00 |
| **Q3** | Soft food sticks to dentures but does not make them uncomfortable | 0(0%) | 0(0%) | 4(19%) | 17(81%) | 0.13 |
| **Q4** | Bacteria tend not to stick to the surfaces of dentures | 1(5%) | 1(5%) | 4(20%) | 16(80%) | 0.38 |
| **Q5** | Unclean dentures can cause mouth infections | 22(100%) | 0(0%) | 0(0%) | 0(0%) | NA |
| **Q6** | For patients comfort dentures should be rinsed after every meal. | 20(100%) | 0(0%) | 0(0%) | 0(0%) | NA |
| **Q7** | Patients with no natural teeth only need check-up when there is a problem | 2(12%) | 3(18%) | 1(6%) | 11(65%) | 0.63 |
| **Q8** | Patients notice discomfort if they have gum infection under dentures | 11(52%) | 2(10%) | 4(19%) | 4(19%) | 0.69 |
| **Q9** | A dirty denture will not cause any disease in the mouth | 0(0%) | 2(10%) | 1(5%) | 17(85%) | 1.00 |
| **Q10** | Brushing cleans dentures more effectively than soaking in denture cleaner | 10(50%) | 7(35%) | 1(5%) | 2(10%) | 0.070 |
| **Q11** | Wearing a denture increases the number of bacteria in the mouth | 5(33%) | 5(33%) | 2(14%) | 3(20%) | 0.45 |
| **Q12** | A softer toothbrush is better than a hard one for cleaning patient's teeth | 11(69%) | 0(0%) | 5(31%) | 0(0%) | 0.063 |
| **Q13** | A large-headed toothbrush is less efficient at cleaning teeth than a small headed one | 7(35%) | 0(0%) | 12(60%) | 1(5%) | <0.001** |
| **Q14** | Lack of calcium can put patients at risk from tooth decay | 11(52%) | 10(48%) | 0(0%) | 0(0%) | <0.001** |
| **Q15** | Old people's teeth are less prone to decay than younger people's teeth | 0(0%) | 1(5%) | 2(10%) | 18(85%) | 1.00 |
| **Q16** | Brushing teeth improves condition of gums | 21(95%) | 0(0%) | 1(5%) | 0(0%) | 0.50 |
| **Q17** | If patients have lots of sugary food and drink their teeth are more likely to decay | 19(90%) | 0(0%) | 2(10%) | 0(0%) | 0.50 |
| **Q18** | A mouth swab is a good alternative to a toothbrush for cleaning patients' teeth | 3(15%) | 1(5%) | 9(45%) | 7(35%) | 0.022* |
| **Q19** | It is possible to catch certain infections from contact with a patient's saliva | 16(89%) | 0(0%) | 2(11%) | 0(0%) | 0.50 |
| **Q20** | Bacteria in patients' mouths are one of the causes of dental decay | 19(90%) | 2(10%) | 0(0%) | 0(0%) | 0.50 |
| **Q21** | Patients with dry mouths will tend to get less decay | 3(14%) | 0(0%) | 18(86%) | 0(0%) | <0.001** |
| **Q22** | Even if the gums around the teeth are inflamed or bleeding they do not usually cause any pain | 3(14%) | 0(0%) | 5(23%) | 14(64%) | 0.063 |
| **Q23** | You should wear protective gloves when cleaning patients' teeth | 19(86%) | 1(5%) | 2(9%) | 0(0%) | 1.00 |
| **Q24** | Most patients with bad teeth will have inherited a tendency to get decay | 3(19%) | 3(19%) | 0(0%) | 10(63%) | 0.25 |
| **Q25** | Once gum disease has started it is almost impossible to halt | 2(12%) | 0(0%) | 1(6%) | 14(82%) | 1.00 |

| **Questions 27-38 (Likert scale)** | | **Before** | | | **After** | | | **After-** |  |
| --- | --- | --- | --- | --- | --- | --- | --- | --- | --- |
|  | | **n** | **Mean** | **SD** | **n** | **Mean** | **SD** | **Before** | **p** |
| **Q27** | I believe my own teeth should last me throughout my life | 23 | 1.91 | 0.79 | 16 | 1.31 | 0.48 | -0.50(0.63) | 0.0064 |
| **Q28** | There is very little I can do to prevent myself getting dental problems | 23 | 3.87 | 1.32 | 16 | 4.19 | 1.17 | 0.19(1.52) | 0.63 |
| **Q29** | Dentures are less trouble than looking after your own teeth | 22 | 4.00 | 1.25 | 16 | 4.06 | 1.12 | 0.13(1.36) | 0.71 |
| **Q30** | If my gums bleed when I brush it means I have been brushing too hard | 23 | 4.04 | 1.07 | 16 | 3.56 | 1.46 | -0.19(1.38) | 0.59 |
| **Q31** | I have looked after my teeth well | 23 | 2.13 | 0.97 | 15 | 1.75 | 0.45 | -0.13(0.62) | 0.43 |
| **Q32** | As you get older you are bound to lose some of your teeth | 23 | 3.13 | 1.18 | 15 | 3.00 | 1.13 | -0.13(1.13) | 0.65 |
| **Q33** | If I was too ill or disabled to clean my own teeth I hope someone would do it for me | 22 | 1.18 | 0.39 | 15 | 1.13 | 0.35 | -0.13(0.52) | 0.33 |
| **Q34** | I worry that I haven't been able to look after my teeth as well as I would have liked | 23 | 2.35 | 1.19 | 15 | 2.73 | 1.28 | 0.13(1.30) | 0.70 |
| **Q35** | It is important for me to keep all of my own teeth | 23 | 1.78 | 0.90 | 15 | 1.40 | 0.51 | -0.33(0.82) | 0.14 |
| **Q36** | I rely on the dentist to prevent me from getting dental problems | 23 | 2.74 | 1.29 | 15 | 2.87 | 1.30 | 0.27(1.53) | 0.51 |
| **Q37** | If my gums bleed when I brush my teeth worry that I am not looking after them well enough | 22 | 2.45 | 0.96 | 11 | 2.45 | 1.13 | -0.27(1.74) | 0.61 |
| **Q38** | It is my responsibility to look after the health of my mouth | 22 | 1.73 | 1.03 | 11 | 1.27 | 0.47 | -0.64(1.21) | 0.11 |

**Key:** T-T = scored true before and true after training; T-F = scored true before and false after training; F-T = scored false before and true after training; F-F = scored false before and false after training. Only the T-F and F-T answers were entered into the statistical analysis using McNemar’s test. NA = no analysis; * = p < 0.05; ** p < 0.001. Questions 27-38 Scored on Likert Scale where 1 = strongly agree; 2 = agree; 3 = neither agree nor disagree; 4 disagree; 5=strongly disagree. n = number of participants; SD = Standard Deviation.

**
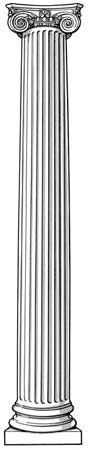
**

***Staff Interview Schedule***

**Stroke**

**Oral Health**

**Care tooL**

**Evaluation (SOCLE)**

**Pilot**

- Perceptions of pilot
  - What worked (e.g. information provision, training, tools, equipment, ward routine/roles, protocol, care planning, patients reactions, onward referrals (MDT/Dental specialists), discharge)
  - What did not work (e.g. information provision, training, tools, equipment, ward routine/roles, protocol, care planning, patients reactions, on-ward referrals (MDT/Dental specialists), discharge)
  - Training
- Quality
  - Do you feel confident assessing whether teeth or dentures are satisfactorily cleaned/identifying problems (cavities, bacteria, fungus…)? Assessing the patient’s ability to perform independent oral care? Frequency of assessment?
  - Liaison with members of the MDT to improve OHC for individual patients….OT, SLT, Physio? Benefits? Problems?
- Gaps
  - For patients with different needs/levels of impairment - e.g. dysphagic patients? paresis of their dominant hand? who are unable to hold a toothbrush or are unable to move the brush effectively around their teeth or dentures? who refuse oral care interventions?
  - How did OHC fit into your daily routine?
  - Time, equipment, knowledge, lack of support from other multidisciplinary team members and senior staff, job descriptions, unwillingness of patients? Any perceived dangers: fear of causing pain/discomfort/bleeding? How confident are you at providing OHC?
- Concordance/deviation from tools
  - How often do you provide these OHC interventions? What are the benefits to patients in providing such care?
  - Inspection of the gums/ tongue / lips / teeth…..
- Adjustments/amendments for future

**
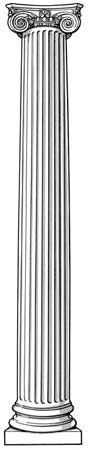
**

**Stroke**

**Oral Health**

**Care tooL**

**Evaluation (SOCLE)**

**Pilot**

***Patient Interview Schedule***

Should the welfare guardian or carer be interviewed in cases where a patient is unable to participate in an interview the grammar will be changed accordingly.

What were your perceptions of your oral health (care for mouth/teeth/dentures)?

- Were you given enough information? (Too little? Too much? Format)

How did the ward routine and caring for your mouth/teeth/dentures fit with your usual routine

- (ward v personal oral health care routine)

How aware were you of the pilot study?

- - (What made you aware of it?)

What aspects of caring for your mouth/teeth/dentures did you think was good?

- - Did you find the process supportive of your OHC

(e.g. information provision, knowledge of nursing staff, availability of equipment, ward routine/roles, consulted about oral health and oral health care).

Which members of staff helped you care for your mouth/teeth/dentures?

- - Staff involvement (nursing, other MDT members/Dental specialists)
  - Did you find the process supportive of your rehabilitation goals
  - Did you find the process supportive of your independence

What (if any) aspects of caring for your mouth/teeth/dentures were you unhappy about?

(Or Which aspects of caring for your mouth/teeth/dentures did you think could have been done better? And How?)

Did you ever feel that your oral health was neglected at any point?

- How important did you think your oral health was?

While in hospital, how important was it for you to care for your mouth/teeth/dentures?

- How important was it for you to be able to **independently** care for your own mouth/teeth/dentures?

Adjustments/amendments for future.
